# Individualized Medicine in Africa: Bringing the Practice Into the Realms of Population Heterogeneity

**DOI:** 10.3389/fgene.2022.853969

**Published:** 2022-04-14

**Authors:** Ayman A. Hussein, Reem Hamad, Melanie J. Newport, Muntaser E. Ibrahim

**Affiliations:** ^1^ Unit of Diseases and Diversity, Department of Molecular Biology, Institute of Endemic Diseases, University of Khartoum, Khartoum, Sudan; ^2^ Department of Global Health and Infection, Brighton and Sussex Medical School, University of Sussex, Brighton, United Kingdom

**Keywords:** individualized medicine, Africa, population heterogeneity, effective size, genetics

## Abstract

The declared aim of “personalized”, “stratified” or “precision” approaches is to place individual variation, as ascertained through genomic and various other biomarkers, at the heart of Scientific Medicine using it to predict risk of disease or response to therapy and to tailor interventions and target therapies so as to maximize benefit and minimize risk for individual patients and efficiency for the health care system overall. It is often contrasted to current practices for which the scientific base is rooted in concepts of a “universal biology” and a “typical” or “average patient” and in which variation is ignored. Yet both approaches equally overlook the hierarchical nature of human variation and the critical importance of differences between populations. Impact of genetic heterogeneity has to be seen within that context to be meaningful and subsequently useful. In Africa such complexity is compounded by the high effective size of its populations, their diverse histories and the diversity of the environmental terrains they occupy, rendering analysis of gene environment interactions including the establishment of phenotype genotype correlations even more cumbersome. Henceforth “Individualized” methods and approaches can only magnify the shortcomings of universal approaches if adopted without due regard to these complexities. In the current perspective we review examples of potential hurdles that may confront biomedical scientists and analysts in genomic medicine in clinical and public health genomics in Africa citing specific examples from the current SARS-COV2 pandemic and the challenges of establishing reference biobanks and pharmacogenomics reference values.

## Background

The concept of individualized medicine invokes some fundamental paradigms relating to human categorization, the attribution to the individual of membership in a group, and to the characterization of variation both within and between groups. The work of the pioneering biometricians, such as Francis Galton, who studied variation between individuals, was often inextricably tied to the beliefs and practices of eugenics which had the expressed aim of “improvement of stock”, “cultivation of race” or some similar formulation of the goal of improving the “genetic quality” of a population. In the period leading up to the Second World War, the misguided adoption of such aims and practices by states in relation to their citizens marred not only eugenics but also to varying degrees its founders and their scientific contributions. In a similar vein, the study of variation between populations and the linked attempts at categorization had descended through scientifically aberrant theories of race to morally aberrant practices of racism and eventually genocide. In contrast, during roughly the same period, the extensive global usage of antibiotics, based in the universal scientific concepts of biology, gave us a lifesaving tool reinforcing the unity of humankind and altering forever our relationship with microbes. The triumphs of antimicrobial drugs, consolidated thereafter by the eradication of smallpox and malaria in the tropics, and the abuse of the concepts of race and eugenics both promoted a holistic humanistic approach to health.

However, the mainstay of the practice of medicine, in the sense of the conventional relationship between the physician and patient, remained individualized. Ironically, individualized and universal approaches have a common thread in that both overlook the stark realities of population heterogeneity. Individuals are genetically defined not through an abstract human category, but through bonds to defined social units like families and populations formed through, among other things, mating and admixture. These units are defined biologically by genetic structure and allele frequency metrics. One of the earliest demonstrations of the relevance of such units to identification and mapping of diseases susceptibility loci is Paul Mckeigue’s work on admixture mapping (Mckeigue, 1998), a concept based on the tenet that population with differential disease phenotype frequencies and recent admixture can help map disease susceptibility loci that are eventually and by nature individualized. Another example is the utility in association studies of alleles imputed from ancestry-adjusted population reference panels, which has been shown to be sensitive to population stratification (Jallow et al., 2009; Huang et al., 2011). Whether stratified, individualized or precise, often used alternately or synonymously, these terms suffer the inherent limitations of definition, for each may describe in different ways the kinds of future medicine and healthcare that these new methods promise to bring forth (Erikainen and Chan, 2018).

It is not only the concepts that await the development of a working paradigm, the practice itself is searching to define its functional domains, including the development of robust and efficient systems for estimation of personalized intervention recommendations as well as the generation of new knowledge about a particular decision process under study (Hicks et al., 2016; Kosorok et al., 2021).

In this perspective we discuss how these paradigms and concepts may translate within the peculiar African setting to describe situations of relevance to its future practice both semantically and functionally.

## Africa’s Burden of Extremes: Environment Interactions and Selection

**FIGURE 1:**
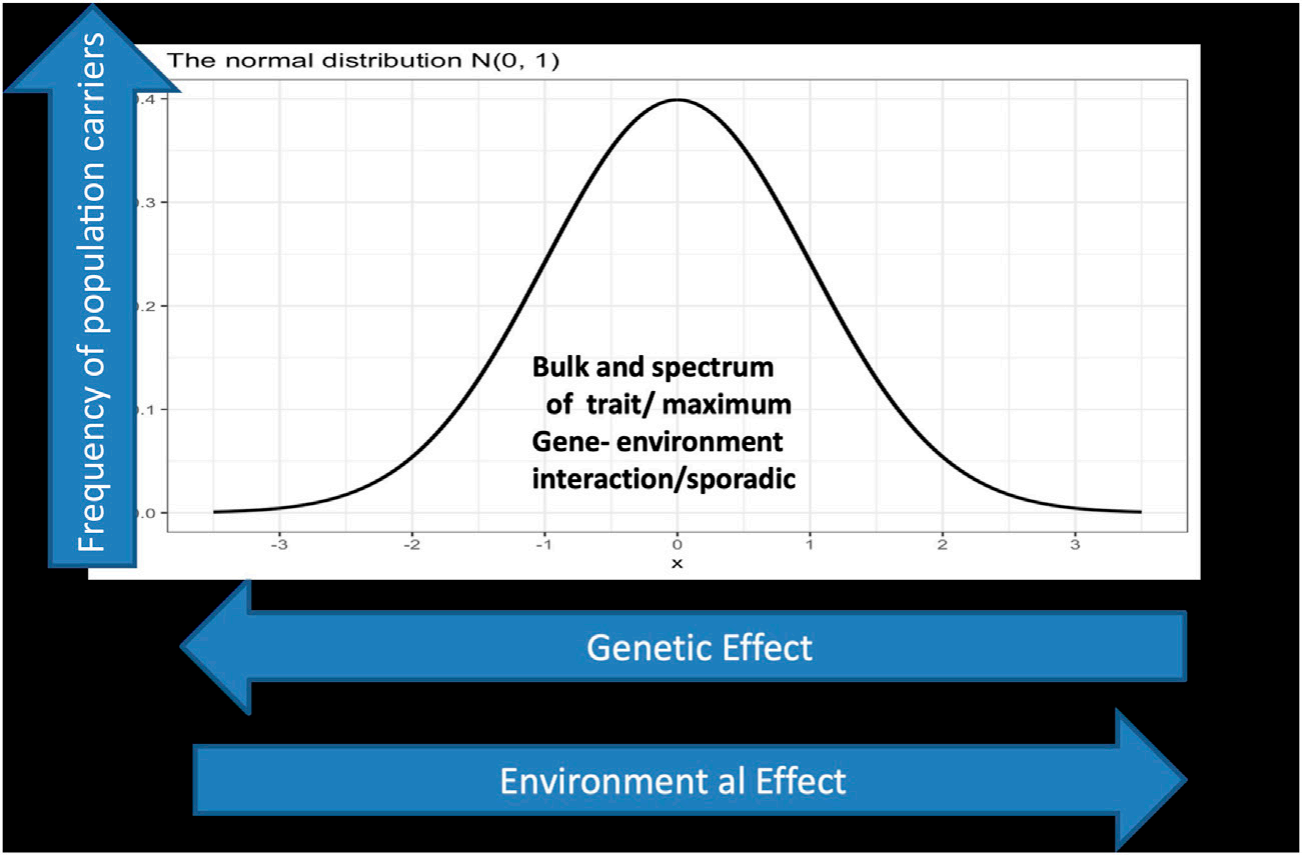
displays a spectrum of gene environment interaction in a two dimensional landscape. The *Y* axis represents the phenotypic outcome of interactions which is normally distributed, while the *x* axis depicts the genetic/environmental effects which is a continuum starting on the left side with large genetic effect as in the case of monogenic and Mendelian inheritance and ends at the right side with low genetic effect but larger environmental effect, in the sense that genetic architecture matters less. Individuals carrying deleterious mutations in their genome with large effects fall under this category which represents in normal situations a tiny fraction of the population. For example a gene that exert strong effect in a Mendelian fashion between 0 and 10 in the *X* axis but has much lower effect in the scale between 90 and 100 in the *Y* axis. In the far right one assumes all the lucky ones who indulge in reckless lifestyle but ends up exceeding the average life span well into their eighties and nineties.

With sequencing of the human genome and the eruption of massive genomic data and knowledge, biological understanding of inheritance progressed from simplistic single gene models into dictums of complex inheritance. The spectrum of interaction from a systems point of view is pivotal. There are conditions where a limited number of variants decide the ultimate phenotype vis-a-vis others that involve multiple variants. It is not only the complexity in the spectrum of gene/gene and gene/protein interactions in a two-dimensional scale as in Figure 1 below; matters may become more complex and multidimensional when bearing in mind that our understanding of genome regulatory mechanisms and the basis of complex inheritance is still in its infancy.

On average, and excluding environmental evolutionary pressure in favor of heterosis, the normal distribution of traits holds. That said, in most situations, population variation remains under the influence of genes with modest or low effects in relation to environmental determinants which constitute the core and essence of complex genetic inheritance. Unfortunately, the prevailing mind-set in the realm of inheritance is largely molded onto single genes with large effects and has little consideration of the compound world of complex and polygenic inheritance i.e., real-life genetics. Ramification of such scope on individualized medicine is consequentially significant. Diagnosticians and therapists should be informed when seeking successful intervention in ailments underpinned by gene-environmental interaction, which form the majority of diseases particularly in our modern lifestyle, characterized by vast and novel psychological and environmental onslaughts on our body and mind. Grappling with such situations is a major undertaking, and genomic science might not be prepared to handle, computationally and conceptually, complex situations of this sort. In Africa, given the large effective population size, its vast and diverse environmental terrains these challenges are even far greater. Ibrahim and Bekele (2019); A Framework for the Implementation of Genomic Medicine for Public Health in Africa: A report by the African Academy of Sciences, 2021).

## The Caveat of African Effective Population Size

Effective Population size (Ne) is a mathematical term denoting the amount of variation in a group, which could be a population, a group of people or even the entire human species. The estimated Ne of Homo sapiens, which describes the number of heterozygous contributing unique genomes at the naissance of our species, is between 1,000 and 10,000 genomes (Elhassan et al., 2014). The majority of this variation is retained in Africa as demonstrated in the spectacular diversity among and within African populations. In fitness terms this may constitute a huge advantage in terms of adaptedness and adaptation, and may have contributed in a historical sense to the survival of our species. However, when it comes to developing therapies and investigations, whether individualized or universal, such heterogeneity can become a liability. In economic and practical terms, the rules of aggregate demand and economies of scale favor large homogeneous populations.

The practice of individualized genomics, though fashionable and tempting, and perhaps within reach in the foreseeable future, may not become commonly applicable in Africa for years to come. Reasons may obviously pertain to Africa’s under-resourced health systems but also to several other innate obstacles, including Africa’s known lower linkage disequilibrium and high genetic diversity (Reich et al., 2001). A major concern questions the argument of a putative ancestral genome that may be reconstructed from existing genomes. If such a premise is flawed progress in translational genomics may not be transferrable in a globalized manner. Two arguments were mentioned along this line: firstly, human populations in and out of Africa were influenced and shaped by drift and serial founder effects (Chiaroni et al., 2009), and secondly, functional variants are multiple, formed through an extended evolutionary history in Africa and retained within its extant populations (Ibrahim and Musa, 2015). It is essentially the same reservation that surrounds the use of genome wide association studies (GWAS) and SNPChips in Africa. There are sobering examples to guard against similar undertakings. Malaria is the most common infectious diseases in Africa, and its phenotypes both as infection or diseases could be readily ascertained. In a GWAS markers close to the sickle cell allele, a locus with a markedly strong effect in protecting against malaria, failed to appear in the initial analysis (minimum *p* = 3.9 × 10^–7^). However, when the sickle allele (rs334) was directly genotyped an impressive signal of *p* = 1.3 × 10^–28^ was recovered. A reference panel of Gambian control DNAs were sequenced across this HHB region and imputation using data derived from this gave a *p* value of *p* = 4.5 × 10^–14^ for the sickle allele (Jallow et al., 2009).

Even if technologies availed a panel of genetic markers with persuasive power for detecting disease association at a reasonable effect size, such markers cannot guarantee satisfactory outcomes in prediction/intervention without accounting for potential epistasis and network effects (Maron et al., 2021), as well as the excess of minor/rare/private pathogenic alleles in Africa (Koko et al., 2018).

## Population Structure in Public Health Genomics, Pharmacogenomics and Biobanking

Africa’s diverse and complex population structure is a vivid reminder to the importance of human genome variation to genomic medicine. It also exposes the feebleness of universal approaches and shows the extent to which management can often fall into uninformed and guess-like practice. As the road towards individualized medicine is still long in Africa, population health maps may be a valuable aid to physicians and diagnosticians. These are now cropping up following genomics mega projects like H3Africa (H3Africa Consortium et al., 2014) and countries/population maps like the recent copy number variation in Tunisian populations (Romdhane et al., 2021).

In many countries the distribution of monogenic diseases is related to ethnic origin or population subgroup and such knowledge is of great assistance. This may similarly apply to polygenic diseases, infectious or non-infectious alike. For monogenic non-infectious diseases, ethnic and geographic clusters are reported at the global, regional and country levels. Similarly, infectious diseases, including the current coronavirus pandemic, can have clear ethnic and geographic distributions (Ibrahim and Salih, 2021). Such clusters may be interrogated for allele frequency patterns and ancestry markers that may foster identification of functional loci and suggest treatments.

Genomic data on such clusters and foci combined with well-documented clinical phenotypic data is expected to improve our understanding of concepts of risk and their measures whether at the level of population, family or individual, and provide clues to why some individuals remain healthy while others are more susceptible to disease. Such knowledge may be used to make predictions about the likelihood that a person will contract a disease and his/her expected clinical phenotype (Molster et al., 2018). It could also be applied to develop new tools for risk prediction or predictive testing in relation to the onset or recurrence of disease, in the context of public health genomics (PHG). PHG is a domain that is most suited to relate populations to both individualized and “universalized” approaches.

Genomic knowledge can offer new ways of stratifying individuals and sub-groups within populations, taking public health beyond the traditional correlates of disease risk factors such as gender, age, and socio-economic status (Boccia et al., 2014). PHG promises wide prospects in informing preventive health measures for all. The P4 (Preventive Proactive, Personalized and Predictive) project, is one such exciting prospect with immense possibilities for harnessing the genomics revolution to achieve the example of health for all (Flores et al., 2013). Pharmacogenomics and pharmacogenetics are largely seen as the domains where PHG could readily inform individualized approaches to be put into practice. Already substantial progress has been made in antihypertensive drugs, by targeted therapy based on renin/aldosterone phenotyping (Akintunde et al., 2017). Among other success stories where the logic was made emphatically on the necessity that African populations begin capturing their own pharmacogenetic SNPs for precision medicine, is the Warfarin case (Ndadza et al., 2019; Ndadza et al., 2021; Muyambo et al., 2022) with several private and population specific polymorphisms identified.

However, apart from these gains, the promise of therapies tailored to individual patients, which is the ultimate aspiration of individualized and precision medicine, again faces the obstacles associated with Africa’s large effective population size, and the potential excess of rare pathogenic variants. It is not only a challenge to patients and doctors; it may also diminish the interest from industry. Potential gaps between discovery and translation and industry are not easy to bridge and companies will be less eager if the market is limited. Alternatively, and until cheaper approaches for translating genomics data into individualized interventions for individual patients is within reach, health systems can make substantial gains by employing population stratification and identification of less rare pathogenic variants that could be typed in patients. Although this is short of ideal where all possible biological interactions are considered, it is still superior to a non-informed administration of drugs in a population. Baker et al. (2017), rightly propose a more systematic characterization of the genetic diversity of African ancestry populations, which might help customize the intervention thus reducing cost and effort.

Biobanks are platforms to obtain, store, manage and distribute large collections of biological samples of defined phenotypes and of marked interest for researchers in human, animal or plant specimens. Given Africa’s reported scale of genomic variation, a major question arises on which representative sample that should be analyzed to capture the true amount of genomic variation present in a country, population or community. Hence, in order to form a perspective of alleles frequency spectrum; the scientific community should invest not only on clinical sequencing efforts, which is becoming the norm for biomedical enterprise, but on population data as well. Such data may be obtained from 1) control samples in association studies, 2) active population surveys and 3) biological samples including tissues from tertiary facilities. Biobanks are by virtue a combination of all the above with the added value of including time series data and data from rare diseases that are seldom encountered in cross sectional surveys and sampling.

## Individualized Medicine in Light of the Pandemic

The current pandemic caused by SARS-CoV-2, came as an anticlimax to public perception of human health following decades of successful eradication of infectious diseases. It embodied the gravity of challenges facing the human species in a globalized and environmentally frail world. Unlike the HIV/AIDS where parts of the continent bore the brunt of its burden, in the current SARS-CoV-2 pandemic a different hitherto unexplained situation prevails, characterized by low disease burden and some of the lowest mortality indices (see Corona world meter). Conventional attributes and explanations are lack of transparency, ailing health systems, young populations (Sofonias and Nkengasong, 2021) and again an oversight of the critical elements of genetic susceptibility and population structure (Pairo-Castineira et al., 2021).

Disparities in the population infection and mortality rates in the COVID-19 pandemic (Ibrahim and Salih, 2021), underscores the need for adaptive strategies where personalized and communal intervention becomes directly proportional to the degree of complexity of its etiological basis including the effect of the environment. Environmental triggers are themselves subject to compatibility with molecular targets which are in turn under influence of allele frequency changes which vary between individuals and population to create a situation of unusual patterns of transmission and epidemicity, where even herd immunity, a central tenet in the control of pandemic, becomes contested (Buss et al., 2021). Vaccination as a measure of quelling pandemics falters between astounding successes like in smallpox eradication, measles and polio vaccines and the modest outcomes in the influenza or dengue virus. Understanding the dynamics of COVID-19 immune responses is not critical only for mass vaccination and attainment of herd immunity but also at the individual level for those who are likely to develop serious adverse side effects from the vaccine. Most of the poor compliance reported in some of the COVID-19 vaccines is related to media coverage of rare serious side effects. Africa’s poor vaccination coverage, however, may be attributable not only to anti-vaccination sentiments and vaccine availability/affordability but possibly also to poor compliance emanating from a communal feeling of lack of threat. Yazdan and Robineau argue In all cases, prediction of such adverse effects and sequel of infection requires a move from “population-based policies” to “individual-centred” measures, at both a public health and patient’s management given the heterogeneity of the clinical presentations of Sars-cov2. https://www.icpermed.eu/en/covid-19_personalised-medicine.php. According to Muyambo et al. (2022), given the “current historical moment of COVID-19 pandemic” with systemic and cardiovascular effects of the virus such move might prove mandatory considering the several private and population specific polymorphisms identified in genes potentially influencing interactions with drugs like warfarin.

## Population Differences and Stratification From the Group to the Person

Although the term population is an appealing one, it can often be quite misleading in actual situations given how complex human history is. We have seen above that ethnic allele frequency maps facilitated the identification of diseases susceptibility loci, in conjunction with imputation (Jallow et al., 2009; Huang et al., 2011).

The challenge of population heterogeneity and hence potential disease propensities has been highlighted in the comprehensive report on genomic medicine by the African Academy of Sciences. That report adopted a widely scientifically acclaimed classification of human populations according to linguistic groups (Chiaroni et al., 2009), with Africa host to four major linguistic families. A panel of 1,327 microsatellite markers further identified 14 ancestral population clusters in Africa that also correlated with self-described ethnicity and shared cultural and/or linguistic properties (Tishkoff et al., 2009).

Such clusters should become the prospective aim and focus of Africa’s genomic endeavors as we zoom in to fill the gap between the populations and individual, the latter being the ultimate goal of precision and individualized medicine.

A vivid example of the pivotally of both obvious and cryptic population structures is visceral leishmaniasis (VL). Ethnic differences were intuitively observed in visceral leishmaniasis (VL) and during the quest to understand the genetic basis of susceptibility to VL, stratification of a target ethnically homogenous population was carried out based on Y chromosome haplogroups. Interestingly, this approach increased the likelihood of linkage to an impressive LOD score of 5.656 defining a susceptibility locus associated with carriers of haplogroup A1b1b2b, indicating the presence of hidden population structure and village-specific genetic lineage possibly due to a founder effect (Miller et al., 2007).

The identification, for purposes of transitioning to personalized risk assessment and intervention, of individualized markers that underlie group and population-based risk is hence not a straight forward path in either stratum, and a marker related to disease risk in one population is not necessarily universal either. Two examples of differences between populations illustrate this point. Firstly, Prdx5 a molecule shown to pose considerable risk for various cancers in Asians, displayed different mode of gene expression and reduced risk in Africans (Sudanese) stressing the need for caution in applying findings about risk association from one population to another (Elamin et al., 2013). The other example concerns apparent differences in the biological etiology of breast cancer between Eritrea and neighboring Sudan as seen in the presence of a marked viral etiology in Sudan and a minor contribution in Eritrea, a contrast that may be related to environmental or genetic causes (Fessahaye et al., 2017).

In Sudan, The field of cancer is rife with similar examples where cancers have shown to present with infrequent mutations in TP53 the notorious tumor suppressor (Masri et al., 2002). However, in many cases, genome and epigenome changes in cancers presented with mutations in other p53 family members like P63 and P73 (Suleiman et al., 2015; Abdallah et al., 2018). BRCA1, BRCA2 and p53 mutations are infrequent in Sudanese breast cancer patients. Epigenetic changes are suggested as alternative mechanisms to account for the minor contribution of genetic alterations in these genes in sporadic and familial breast cancer cases in Sudan, and evidence for a strong association with EBV was reported (Yahia et al., 2014). These findings were subsequently corroborated by whole methylome analysis which pointed out the dysregulation of specific developmental and viral pathways (Abdallah et al., 2018). In the progression of hereditary colorectal cancer, emphasis is usually placed on the pathogenic role of inactivating mutations in tumor-suppressor genes; however, a study of a multi-case family revealed a different pathway of similar complexity with the identification of a pivotal role for the RNA binding protein ELAV1 and other partners (Suleiman et al., 2015). The presence or absence of a variant is not necessarily a direct reflection of the absence or presence of a trait. An interesting example is lactase persistence. In Europe a single mutation associated with lactase persistence dominates, whereas at least 4 mutations associated with the persistence of the enzyme, mainly in milk-consuming pastoralists communities, were identified in Africa (Ranciaro et al., 2014). However, more than one population that has a history of dairy consumption has no obvious mutations in lactase gene to explain the trait. Apparently milk digestion and lactose tolerance may not be solely an outcome of genetic adaptation. Speculation points to lactobacilli possibly underlying the trait. If this is the case either directly or epigenetically, another layer of variation has to be taken in to account which is the role of the microbiome.

Beyond conventional paradigms of gene-gene interaction, where many gray areas still exists, defining gene-environment interaction is also exceedingly challenging in Africa for the same reasons: These complex interactions between environmental determinants (viruses, food etc.), genomic sequences and epigenetic machinery is what renders the pursuits of individualized approaches in Africa a daunting task but all more interesting. Amid the mist of these disparate and multitude of contributors one should possess a methodological compass to point the right direction where the phenotypic outcome are plausibly explained by genetic variants shown in population differences, while environmental and epigenetic determinants need to be worked out in a frame work of system science. (Kosorok et al., 2021; Maron et al., 2021). As the aim of precision medicine is to design and improve diagnosis, therapeutics and prognostication through the use of large complex datasets that incorporate individual gene, functional, and environmental variations. The implementation of high-performance computing (HPC) and artificial intelligence (AI) can predict risks with greater accuracy based on available multidimensional clinical and biological datasets(Hicks et al., 2016; Subramanian et al., 2020; Kosorok et al., 2021).

In conclusion the road towards an effective, equitable and cost-efficient personalized health practices in Africa still has a long way to go. Major obstacles include the complex phenotypic outcomes of gene-environment interactions, the difficulty of applying customized approaches in an environment of remarkable genetic diversity, the low translational value of genetic and genomic research, prohibitive cost of technologies and applications, and availability of these applications to the bulk of Africans and finally the disinterest of state and business. For Africa to enter the epoch of individualized medicine will not be possible without addressing these challenges and carrying out fundamental reform to integrate genomics into the educational and health system.

## Data Availability

The original contributions presented in the study are included in the article/Supplementary Material, further inquiries can be directed to the corresponding author.
